# Supplementary material for: Chirality manipulation of ultrafast phase switches in a correlated CDW-Weyl semimetal
Source: Nat Commun. 2024 Jan 26;15:785. doi: 10.1038/s41467-024-45036-1 (PMC10817907; doi:10.1038/s41467-024-45036-1)
Supplement: Supplementary file 1 — SUPPLEMENTARY INFO [file 41467_2024_45036_MOESM1_ESM.pdf]

Supplementary Information: Chirality manipulation of ultrafast phase switches in a correlated CDW-Weyl semimetal

Bing Cheng,[1,*] Di Cheng,[1] Tao Jiang,[1] Wei Xia,[2,3] Boqun Song,[1,4] Martin Mootz,[4] Liang Luo,[1] Ilias E. Perakis,[5] Yongxin Yao,[1] Yanfeng Guo,[2,3] and Jigang Wang[1,4,†]

[1]*Ames National Laboratory, Ames, IA 50011 USA*
[2]*School of Physical Science and Technology, ShanghaiTech University, Shanghai 201210, China*
[3]*ShanghaiTech Laboratory for Topological Physics, Shanghai 201210, China*
[4]*Department of Physics and Astronomy, Iowa State University, Ames, Iowa 50011, USA.*
[5]*Department of Physics, University of Alabama at Birmingham, Birmingham, AL 35294-1170, USA.*
(Dated: January 8, 2024)

Supplementary Note 1: Sample characterization

The phase and sample quality of $(TaSe_4)_2I$ were examined on a Bruker D8 single-crystal x-ray diffractometer with Mo $K_{\alpha 1}$ ($\lambda = 0.71073$ $\mathring{A}$) at 298 K. The diffraction pattern as shown in Supplementary Fig. 1(a)-1(c) can be well indexed on the basis of a body-centered tetragonal structure with lattice parameters $a = 9.53530$ A, $b = 9.53530$ A, $c = 12.77770$ A, $\alpha = 90°$, $\beta = 90°$, and $\gamma = 90°$ in the space group 97 ($I422$), consistent with previous reports[1]. The perfect reciprocal lattice space without any other miscellaneous points indicates the crystal is of high quality. The dc resistivity along the chain direction (c axis) is displayed in Supplementary Fig. 1(d). The resistivity shows a typically insulating behavior at low temperature and develops a weak kink near $T_{\mathbf{CDW}} \sim 260$ K. These features are consistent with the scenario of an insulating CDW ground state and a CDW phase transition near 260 K[2, 3].

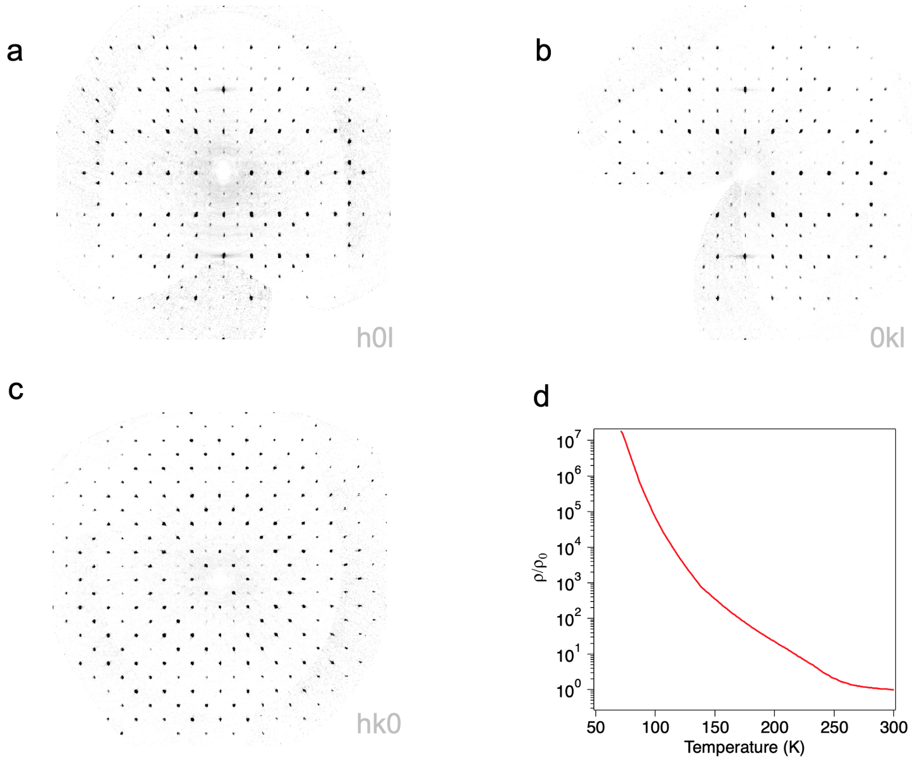

Supplementary Fig. 1: Diffraction patterns in the reciprocal space along the (a) (h 0 l), (b) (0 k l), and (c) (h k 0) directions. (d) The temperature dependent dc resistivity of $(TaSe_4)_2I$ single crystal. The current is set along the chain (c axis) direction. The resistivity is normalized by the room temperature resistivity ρ_0.

Supplementary Note 2: Full fluence dependent THz photocurrent emission data at 5 k

In the main text, we discussed the fluence dependence of THz emission from the longitudinal circular photogalvanic photocurrent (L-CPGE) at 5 K. Here we show full fluence dependent photocurrent emission data at 5 K. The measurement geometry is shown in main text Fig. 2(a). From the raw dataset, we could see clearly that the L-CPGE current becomes more siginficant as the circularly polarized pump increases. We want to point out that the time trace of THz emission under the fluence of 0.2 mJ/cm^2 shows a little anomaly after 2 ps comparing to the data under other fluences. This anomaly probably comes from the experimental uncertainties. Considering the time trace of L-CPGE component $(E_{\circlearrowright}-E_{\circlearrowleft})/2$ (see main text Fig. 3(b)) dominates before 2 ps, we still use such data as a systematic study.

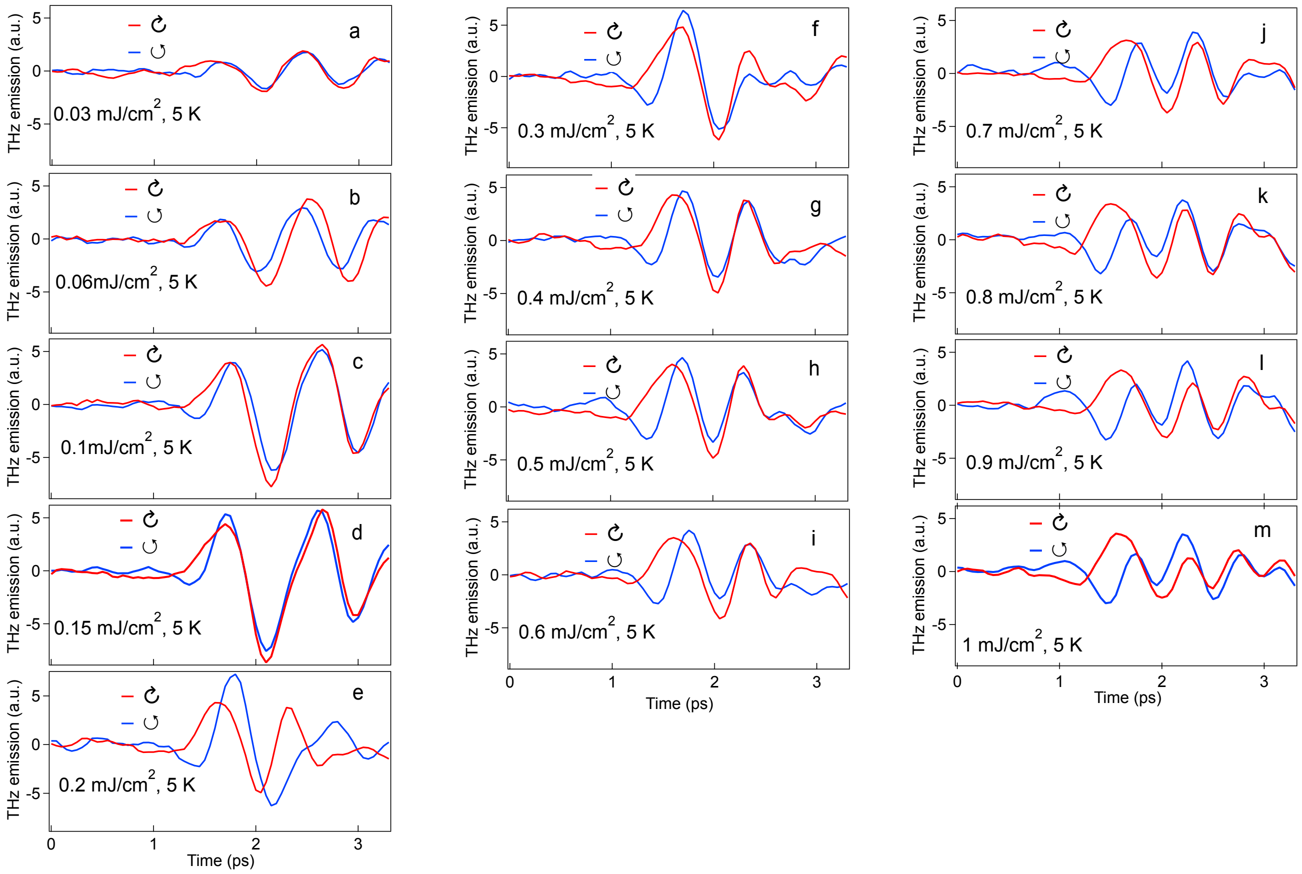

Supplementary Fig. 2: THz photocurrent emission in the longitudinal measurement geometry under circularly polarized pump fluence of (a) 0.03 mJ/cm^2, (b) 0.06 mJ/cm^2, (c) 0.1 mJ/cm^2, (d) 0.15 mJ/cm^2, (e) 0.2 mJ/cm^2, (f) 0.3 mJ/cm^2, (g) 0.4 mJ/cm^2, (h) 0.5 mJ/cm^2, (i) 0.6 mJ/cm^2, (j) 0.7 mJ/cm^2, (k) 0.8 mJ/cm^2, (l) 0.9 mJ/cm^2, (m) 1 mJ/cm^2.

Supplementary Note 3: Full fluence dependent THz photocurrent emission data at 295 k

In the main text, we discussed the fluence dependence of THz emission from the longitudinal circular photogalvanic photocurrent at 295 K. Here we show full fluence dependent photocurrent emission data at 295 K. The measurement geometry is shown in main text Fig. 2(a). In Supplementary Fig. 3(g), we show THz-emission time trace of L-CPGE component $(E_{\circlearrowright}-E_{\circlearrowleft})/2$ under circularly polarized pump fluence of 0.1 to 1 mJ/cm^2. In constrast with the data at 5 K (see main text Fig. 3(b)), the L-CPGE photocurrent increases much more slowly in the high-temperature normal state.

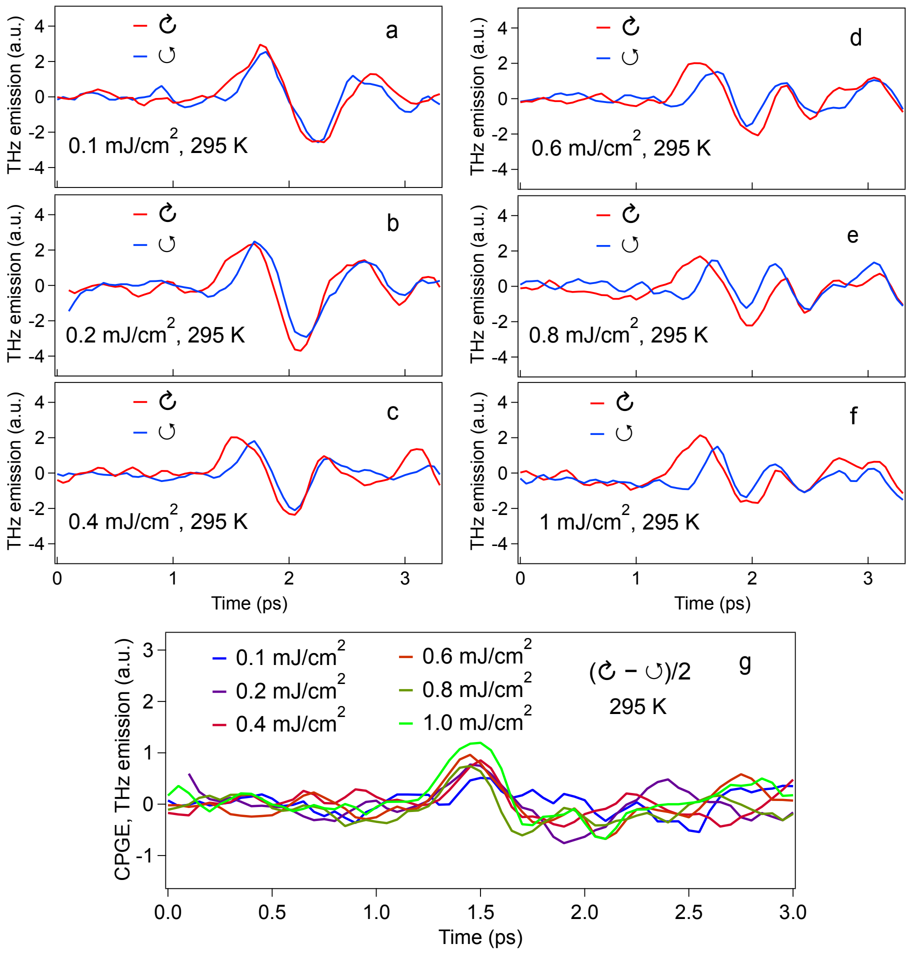

Supplementary Fig. 3: THz photocurrent emission in the longitudinal measurement geometry under circularly polarized pump fluence of (a) 0.1 mJ/cm^2, (b) 0.2 mJ/cm^2, (c) 0.4 mJ/cm^2, (d) 0.6 mJ/cm^2, (e) 0.8 mJ/cm^2, (f) 1 mJ/cm^2. (g) THz-emission time trace of L-CPGE component $(E_{\circlearrowright}-E_{\circlearrowleft})/2$ under pump fluence of 0.1 to 1 mJ/cm^2 at 295 K.

Supplementary Note 4: Some other THz generation mechanisms and their relevance to $(TaSe_4)_2I$

Besides the longitudinal circular photogalvanic effect, other mechanisms, such as the photo-Dember effect, photon drag effect and photo-thermoelectric effect, could be able to contribute THz photocurrent emission too. With further measurements, we could exclude all these sources and underpin the main contribution from the longitudinal circular photogalvanic effect. THz emission may arise from the photon drag effect not involving material symmetry-breaking[4]. To exclude such an effect, one should measure THz emission with normal incident pump and see whether the THz emission signal is negligible or notable in this geometry. If the THz emission is only from the photon drag effect, one should observe THz emission signal with oblique incident pump and negligible THz emission signal with normal incident pump. Our THz emission data with normal incident pump, shown in Supplementary Fig. 4 below, however, is distinctly different from the work[4]. The amplitude of the THz pulse generated with normal incident pump in our case (blue curve, Supplementary Fig. 4) is comparable to that with 45 deg oblique incident pump (red curve, Supplementary Fig. 4), which cannot be explained by the photon drag effect. Moreover, our pump fluence dependent CPGE effect as presented in Fig. 3a of our main text shows a clear linear to non-linear saturation, which cannot be explained by the photon drag effect either. Therefore, the photon drag effect can be safely ruled out in our case. Additionally, the photo-Dember effect and photo-thermoelectric effect can be ruled out too. They both are pump polarization independent and is directed perpendicular to the sample surface, so that the normal incidence geometry cannot measure it[5, 6].

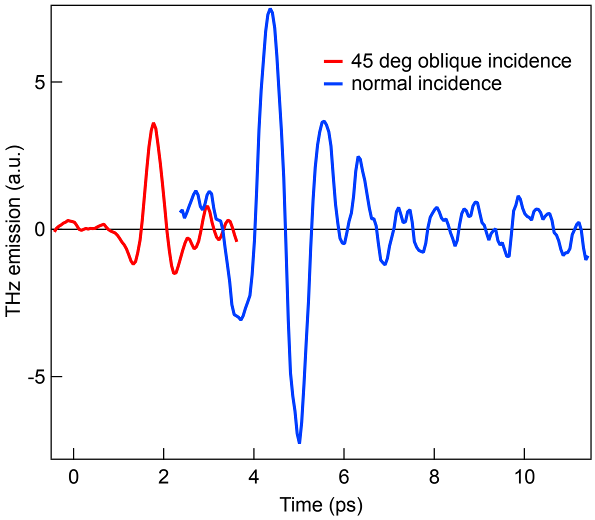

Supplementary Fig. 4: Comparison of THz emission signal with 45 deg oblique incident (red) and normal incident (blue) pump. Note that the former is measured in reflection geometry and the latter is measured in transmission geometry. To achieve a more precise comparison of the THz generation amplitudes, the latter is scaled by the THz transmission coefficient through the sample to account for the additional absorption of THz signal by the sample. The other measurement conditions are the same, e.g., 800 nm pump with vertical polarization, THz emission with polarization perpendicular to the chain, 0.6 mJ/cm^2 fluence, and T = 5 K.

Supplementary Note 5: Full fluence dependent THz phonon emission data at 5 k

As we discussed in the main text, besides the large photocurrent emission from L-CPGE, the THz emission from $(TaSe_4)_2I$ crystal includes a significant phonon emission component. In Supplementary Fig. 5(a), we show the long-time scans of phonon emission under pump fluence of 0.1 to 1 mJ/cm^2. The linearly polarized excitation laser pulse is set perpendicular to the chain. The THz emission is probed in the geometry shown in main text Fig. 2(a). One can see that at high fluence, the phonon emission component develops multi-cycle long-lasting oscillations and beatings in the subsequent extended tens of ps time scale, which indicates the phonon emission contains multiple phonon modes with very close frequencies. The relevant fast Fourier transformation magnitude of the phonon emission is shown in Supplementary Fig. 5(b). Three phonon modes centered at $\sim$ 2.2 THz can be clearly resolved. As the fluence is decreased, the beating feature becomes weak. At 0.1 mJ/cm^2, the beating feature is absent on the phonon emission spectra.

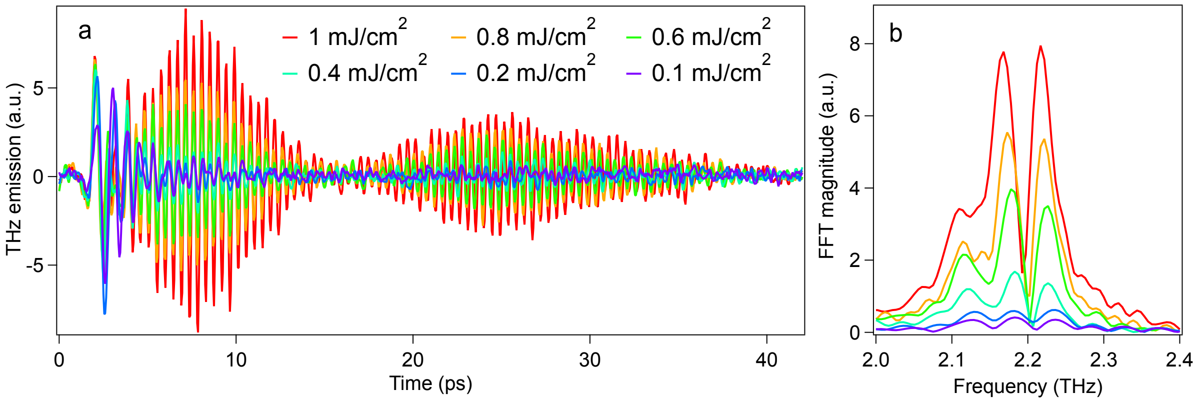

Supplementary Fig. 5: (a) Long-time scan of THz phonon emission in the longitudinal measurement geometry under pump fluence of 0.1 to 1 mJ/cm^2. (b) The Fourier transformation magnitude of the phonon emission component.

To investigate the phonon dynamics and well capture the weak signals of THz phonon emission in the ultralow pump fluence regime, we perform the time scans of phonon emission with less time length. We average more to achieve high statistics. In Supplementary Fig. 6(a), we show the time trace data of the phonon emission under the pump fluence of 0.03 to 0.2 mJ/cm^2. The relevant fast Fourier transformation magnitude of the phonon emission is shown in Supplementary Fig. 6(b). From the time trace and Fourier transformation data at low fluence, we observe a clear crossover from one phonon emission (1.3 THz) dominant at ultralow pump fluence like 0.06 mJ/cm^2 to other phonon emissions ($\sim$ 2.2 THz) dominant at relatively moderate pump fluence like 0.2 mJ/cm^2.

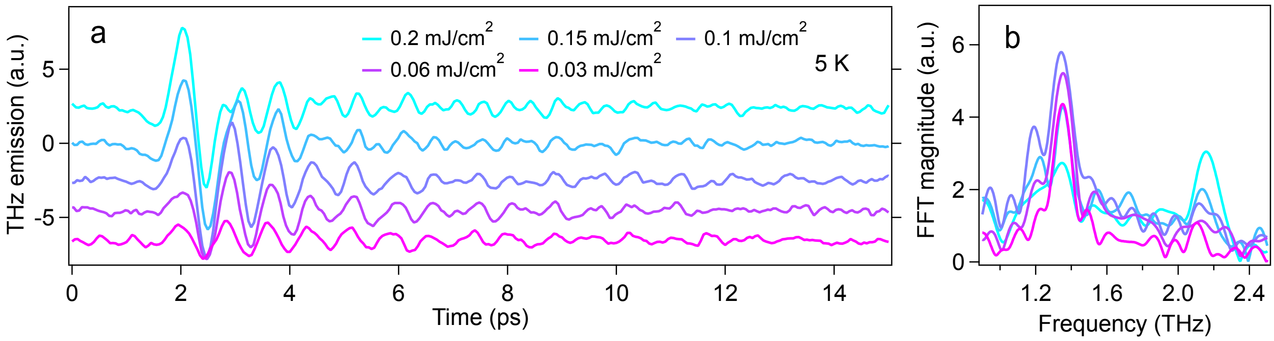

Supplementary Fig. 6: (a) Time tracee of THz phonon emission in the longitudinal measurement geometry under pump fluence of 0.03 to 0.2 mJ/cm^2. (b) The Fourier transformation magnitude of the phonon emission component.

Supplementary Note 6: Full temperature dependent THz phonon emission data

To look for more evidence for the relevance of the phonon emission to the charge-density-wave phase transition, we perform temperature dependent study of the phonon emission. The pump fluence is set to 0.8 mJ/cm^2. The linearly polarized excitation laser pulse is set perpendicular to the chain. The THz emission is probed in the geometry shown in main text Fig. 2(a). The time trace and the corresponding fast Fourier transformation magnitude data are displayed in Supplementary Fig. 7(a) and 7(b) respectively. As temperature increases, the magnitude of phonon emission decays. Most importantly, across $\sim$ 200 K, the phonon emission magnitude is significantly decreased. At the same time, the phonon modes become overdamped. We plot the frequency of phonon modes #2 and #3 as a function of temperature in Supplementary Fig. 7(c). We do not show temperature dependence of phonon mode #1 because its spectral weight is relatively small which makes it is difficult to capture its temperature dependence accurately. One could see, as temperature increases, both phonon modes get softening. Across $T_{\mathbf{CDW}} \sim$ 260 K, the mode frequencies show kinks and do not approach zero. These features strongly indicate the modes revealed by our THz phonon emission data are the lattice modulation-induced zone-folding IR phonons. Our IR phonon spectra calculations based on density functional theory confirm that these phonons are IR B_3 phonons and CDW-related.

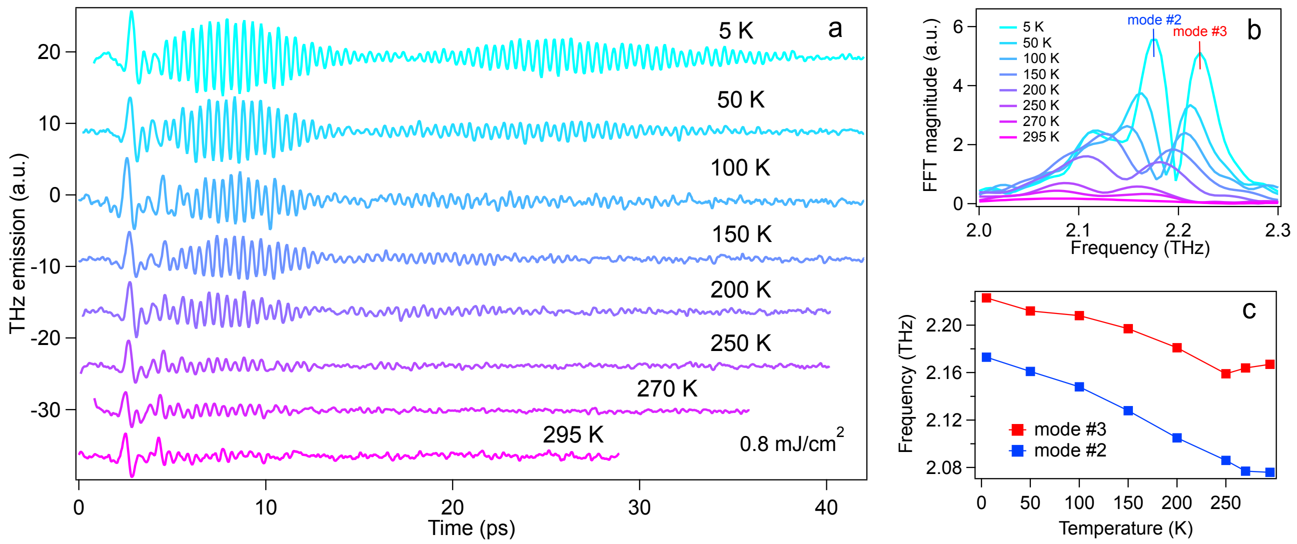

Supplementary Fig. 7: (a) Time tracee of THz phonon emission in the longitudinal measurement geometry at temperature of 5 K to 295 K. The pump fluence is set to 0.8 mJ/cm^2. (b) The Fourier transformation magnitude of the phonon emission component. (c) The frequencies of phonon mode #2 and #3 as a function of temperature. The positions of mode #2 and #3 are labelled in Fig. 7(b).

Supplementary Note 7: The THz detection bandwidth of our setup

We use a laser system with 800 nm central wavelength, 1 kHz repetition rate and 40 fs pulse duration to generate and detect THz pulse. Now we consider the bandwidth realized by our THz laser system. Here, the THz pulse is first generated by a ZnTe crystal, and then goes through a 1 mm pinhole, before it is finally is detected by a second ZnTe crystal. The waveform and the corresponding FFT spectra of the THz pulse are shown in Supplementary Fig. 8(a) and (b) respectively. We can see that our THz detection bandwidth is optimized between 0.5 to 2.5 THz. Therefore, it is not surprising to see that our THz system does not resolve any 0.1 or 0.2 THz modes in $(TaSe_4)_2I$ since the latter is outside of our detection bandwidth.

In addition, we emphasize that our work shows a bunch of zone-folding phonons which are related to the lattice distortion. These zone-folding phonons are observed near 2.2 THz, which are well within our THz detection bandwidth. Actually, a previous optical pump-probe work on $(TaSe_4)_2I$ shows several Raman-active modes related to the CDW phase transition, such as the modes at 0.1, 0.2, 1.1, 2.3, and 2.65 THz[7]. Note that this is a pump-probe experiment that has a suitable geometry and time resolution to be able to observe all these modes, from super-low to high frequency. The symmetry analysis of the lattice structure indicates that these Raman modes should have corresponding infrared-active counterparts at similar frequencies[7]. Our work indeed observes the infrared-active counterparts of these Raman-active modes (at 1.1THz and 2.3 THz) which fall within our detection bandwidth.

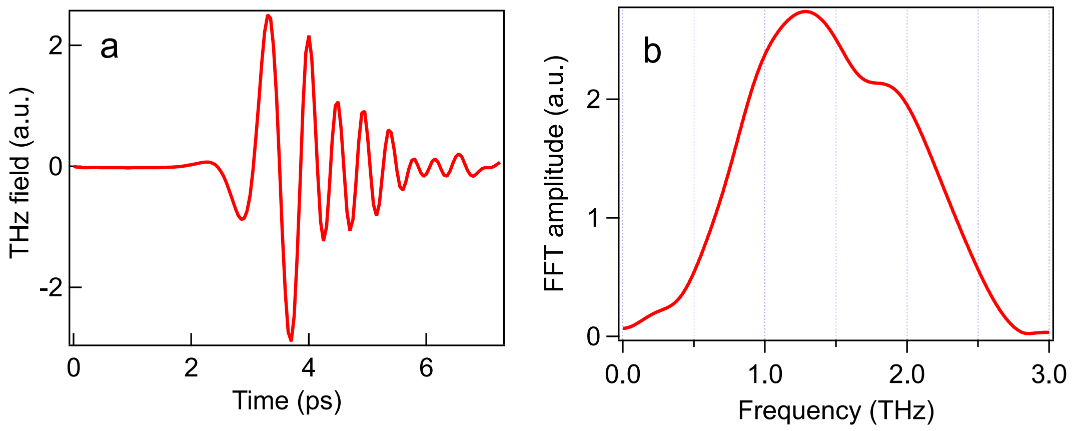

Supplementary Fig. 8: THz waveform (a) and detection bandwidth (b) achieved by using our laser system.

Supplementary Note 8: Crystal structure with phonon eigenvector

We carry out phonon calculations based on density functional theory (DFT) to provide theoretical support for IR modes of $(TaSe_4)_2I$. The non-CDW phase is in the crystal structure with space group $I422$(No.97), and the CDW phase is with space group $F222$(No.22)[8]. For the CDW phase, the IR phonon mode near 2.2 THz has B_3 symmetry. Near 1.3 THz, we identify two almost degenerate IR modes, with B_2 and B_1 symmetry.

In the conventional cell (see Supplementary Fig. 9), there are two Ta atom chains along the c axis surrounded by 4 layers of 16 Se atoms like screws. The B_2 mode near 1.3 THz, as indicated by the arrows, is mainly I_a atom displacing along a-axis while I_b atom displacing along b-axis of the crystal, with small displacements contributed from Ta atoms and Se atoms (see Supplementary Fig. 9 (a)(b)(c)). We use the lattice distortion factor λ to quantify the magnitude of the atomic displacement according to this B_2 mode. For $\lambda = 1.0$, the maximal atomic displacement is 0.058 $\mathring{A}$ for I_a.

For B_3 IR phonon mode near 2.2 THz, as indicated by the arrows, one of its main displacements is Se atoms rotating around the chain (c-axis). In order to show the rotating motion clearly, we use red arrows to represent the phonon eigenvector components of the 1st and 2nd layers, and use blue color to represent that of the 3rd and 4th layers (see Supplementary Fig. 9(d-f)). Along a single chain, the Se atoms of the 1st and 2nd layers rotate in opposite direction against that of the 3rd and 4th layers. The Se atoms in the same layer of nearby chains also rotate in opposite direction, like "gears engagement". Another main displacement component is Ta vibration pattern which we discussed in main text. For $\lambda = 1.0$, the maximal corresponding atomic displacement is 0.034 $\mathring{A}$ for Se_a.

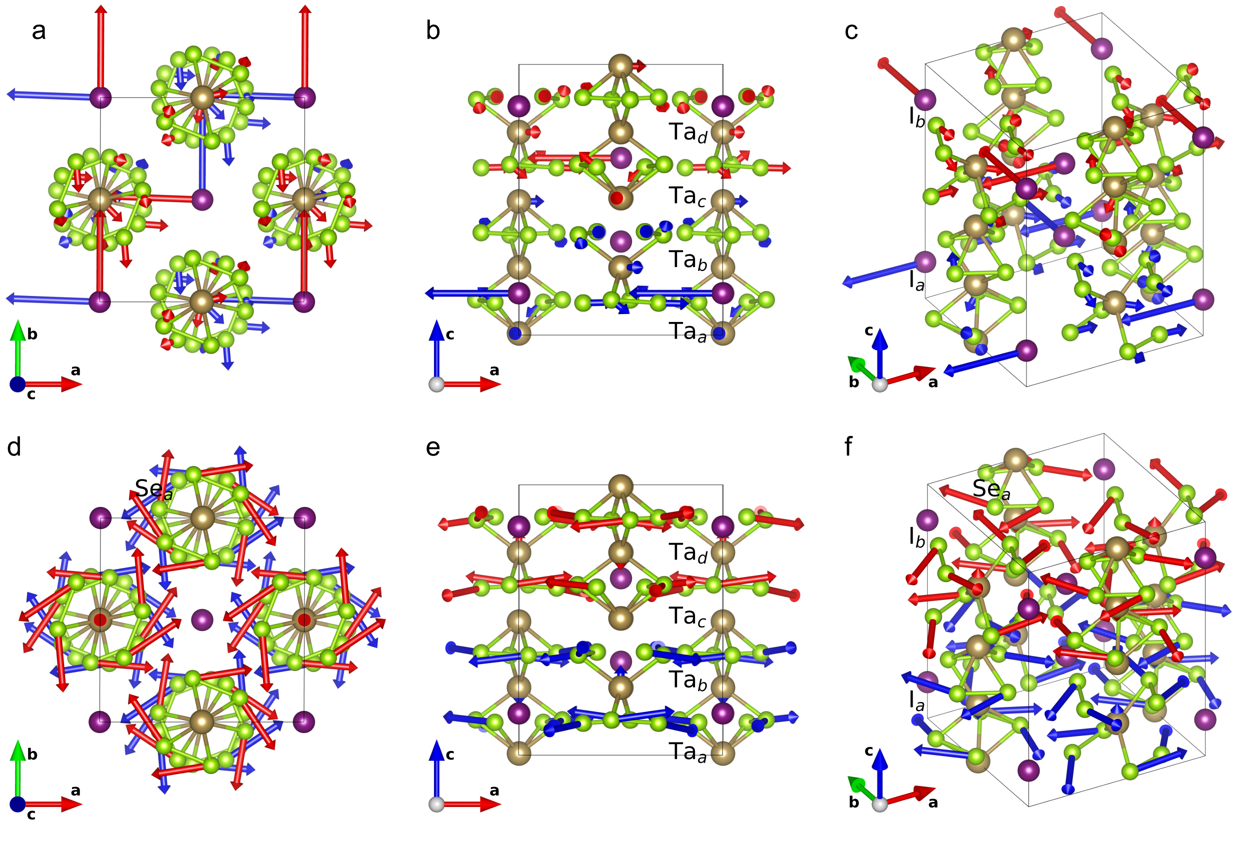

Supplementary Fig. 9: The crystal structure with arrows indicating B_2 IR phonon mode near 1.3 THz (a-c) and B_3 IR mode near 2.2 THz (d-f) are shown from top (001) view, side (010) view, and tilted side view, respectively. The atom types of $(TaSe_4)_2I$ are with the following colors: golden, Ta; green, Se; purple, I. The arrows indicate the direction and magnitude of the atomic displacements of the corresponding phonon mode. Red arrows are for the 1st and 2nd layer and blue arrows are for the 3rd and 4th layer.

Supplementary Note 9: IR phonon intensity

In order to compare the intensity of IR phonon near 1.3 THz for non-CDW phase and CDW phase. We used Phonopy-Spectroscopy package to calculate the IR intensity[?]. For a phonon mode s, the intensity is calculated as:

$$I_{\mathrm{IR}}(s) = \sum_{\alpha=1}^{3} \left| \sum_{j=1}^{n_a} \sum_{\beta=1}^{3} Z_{\alpha\beta}{}^{*}(j) X_{\beta}(s,j) \right|^2 \tag{1}$$

where $Z_{\alpha\beta}{}^{*}(j)$ is the Born effective-charge for the jth atom in the unit cell. And α labels the direction of the macroscopic polarization. n_a is the number of atoms per unit cell. β is the direction for a cooperative displacement of atoms. X_β(s,j) is defined as the phonon eigenvector divided by the square root of the atomic masses.

The results are shown in Table S1, for the double-degenerated IR phonon modes with E symmetry in non-CDW phase, the IR intensity is 0.11 and 0.12 e^2amu^{-1}. In the CDW phase, the intensity of IR B_2 near 1.3 THz is 0.16 e^2amu^{-1}. And the intensity of the nearly degenerate IR B_1 is 0.08 e^2amu^{-1}. Therefore, the total IR intensity near 1.3 THz is 0.24 e^2amu^{-1} in CDW phase, which is slightly larger than 0.23 e^2amu^{-1} in the non-CDW phase.

Supplementary Table 1: Phonon modes of $(TaSe_4)_2I$ (amu is atomic mass unit)

Phase	Frequency (THz)	Irreducible Label	Optical Activity	I_{IR} (e^2amu^{-1})
non-CDW	1.31	E	IR	0.11
non-CDW	1.31	E	IR	0.12
CDW	1.28	B_2	IR	0.16
CDW	1.29	B_1	IR	0.08

Supplementary Note 10: Axion insulator phase in the CDW-Weyl semimetal

The band structure calculation of $(TaSe_4)_2I$ has identified 24 pairs of Weyl points (WPs) in the bulk within 15 meV of the Fermi energy[3]. According to the information of the coordinates of these WPs in momentum space, one can calculate the independent nesting vectors between WPs with opposite chiral charges. X-ray diffraction experiment can directly measure the direction and magnitude of the CDW modulation vectors[3]. By comparing the nesting vectors with the CDW wave vector determined by X-ray diffraction, one can see all of the nesting vectors between the WPs with opposite chiral charges match integer multiples of the experimentally-observed CDW modulation basis vectors[3]. Therefore, all of the WPs with opposite chiral charges can be nested by the experimentally observed CDW modulation vectors, leading to a gapped CDW phase[3].

As the CDW order emerges to gap the Weyl nodes, the Weyl fermions near the nodes becomes massive. Most importantly, two new collective modes, i.e. amplitude mode and phase mode, of CDW order parameter $\Delta e^{i\theta}$ will emerge. Formally, this phase mode θ is termed as axion field or axion mode. It is coupled to the electromagnetic field in the form of θE·B, which has the same form of the axion in the high-energy physics coupling to the electromagnetic field[2, 9]. As a result, the CDW-Weyl semimetal below CDW transition temperature is a correlated axion insulator. One significant effect of this CDW-based axion insulator is the emergence of the chiral-anomaly like current if a parallel electric field E and magnetic field B is applied to this correlated axion insulator. This chiral anomaly effect was subsequently demonstrated by a recent magneto dc transport measurement of $(TaSe_4)_2I$[2]. Despite these exciting progresses, we would like to mention that another dc transport measurement of $(TaSe_4)_2I$ published recently claimed the absence of the chiral-anomaly related negative magneto longitudinal resistivity in $(TaSe_4)_2I$ [10], which raises concerns on the existence of axion insulator phase in $(TaSe_4)_2I$. As discussed in our work, this debate on if $(TaSe_4)_2I$ is a true axion insulator based on dc magneto-transport measurements may arise from the ubiquitous polaron physics in $(TaSe_4)_2I$[2, 10].

* Electronic address: bcheng2@ameslab.gov
† Electronic address: jgwang@ameslab.gov

[1] Tournier-Colletta, C. *et al.* Electronic instability in a zero-gap semiconductor: The charge-density wave in $(TaSe_4)_2I$. *Phys. Rev. Lett.* **110**, 236401 (2013).
[2] Gooth, J. *et al.* Axionic charge-density wave in the Weyl semimetal $(TaSe_4)_2I$. *Nature* **757**, 315 (2019).
[3] Shi, W. *et al.* A charge-density-wave topological semimetal. *Nat. Phys.* **17**, 381 (2021).
[4] Cheng, L. *et al.* Giant photon momentum locked THz emission in a centrosymmetric Dirac semimetal. *Science Advances* **9**, eadd7856 (2023).
[5] Huang, Y. *et al.* Terahertz surface and interface emission spectroscopy for advanced materials. *Journal of Physics: Condensed Matter* **31**, 153001 (2019).
[6] Lu, W. *et al.* Ultrafast photothermoelectric effect in Dirac semimetallic Cd_3As_2 revealed by terahertz emission. *Nat. Commun.* **13**, 1623 (2022).
[7] Schaefer, H. *et al.* Dynamics of charge density wave order in the quasi one dimensional conductor $(TaSe_4)_2I$ probed by femtosecond optical spectroscopy. *The European Physical Journal Special Topics* **222**, 1005 (2013).
[8] Zhang, Y., Lin, L.-F., Moreo, A., Dong, S. & Dagotto, E. First-principles study of the low-temperature charge density wave phase in the quasi-one-dimensional Weyl chiral compound $(TaSe_4)_2I$. *Phys. Rev. B* **101**, 174106 (2020).
[9] Wang, Z. & Zhang, S.-C. Chiral anomaly, charge density waves, and axion strings from Weyl semimetals. *Phys. Rev. B* **87**, 161107 (2013).
[10] Sinchenko, A. A., Ballou, R., Lorenzo, J. E., Grenet, T. & Monceau, P. Does $(TaSe_4)_2I$ really harbor an axionic charge density wave? *Applied Physics Letters* **120**, 063102 (2022).
